# 1408. Epidemiology of Nitroimidazole-refractory Giardiasis

**DOI:** 10.1093/ofid/ofad500.1245

**Published:** 2023-11-27

**Authors:** Karin Ydsten, Joanna Nederby Öhd, Urban Hellgren, Hilmir Asgeirsson

**Affiliations:** S:t Görans Hospital, Stockholm, Stockholms Lan, Sweden; Department of Communicable Disease Control and Prevention, Stockholm County Council, Stockholm, Stockholms Lan, Sweden; Karolinska University Hospital, Stockholm, Stockholms Lan, Sweden; Karolinska University Hospital, Stockholm, Stockholms Lan, Sweden

## Abstract

**Background:**

*Giardia intestinalis (G. lamblia* or *G. duodenalis)* is one of the most common pathogenic intestinal parasitic protozoa. Giardiasis refractory (resistant) to nitroimidazoles (e.g., metronidazole and tinidazole) has increasingly been reported, most notably noticed in travellers returning from the Indian subcontinent. Information on the proportion of nitroimidazole-refractory cases from different world regions is scarce, and is difficult to investigate in a high-endemic setting.

**Methods:**

All reported cases of giardiasis in Stockholm County (population 2,391,990 as of Dec 31^st^ 2020) between January 2008 and December 2020 were obtained from the Department of Communicable Disease Control and Prevention, as it is a notifiable disease in Sweden. Refractory cases in Stockholm are referred to the Karolinska University Hospital, a tertiary centre, for treatment since second line drugs are not easily available. Medical records were reviewed. Nitroimidazole-refractory giardiasis was defined as continued symptoms and positive stool sample for giardiasis (microscopy and/or PCR) after a full treatment course.

**Results:**

During the study period 4285 cases of giardiasis were notified and 95 of those (2.2%) were identified as nitroimidazole-refractory. The median age was 38 years (range 5-78) in the refractory group and 30 years (range 0-89) in the non-refractory. In both groups 55% were males. In those who had acquired the infection in India the refractory rate was 11% (60/545) compared to 1.1% in rest of Asia, 1.3% (14/1113) in Africa, 0.9% (11/1247) in Europe, 0.3% (1/349) in the Americas, and 0% (0/5) in Oceania.

**Figure:**
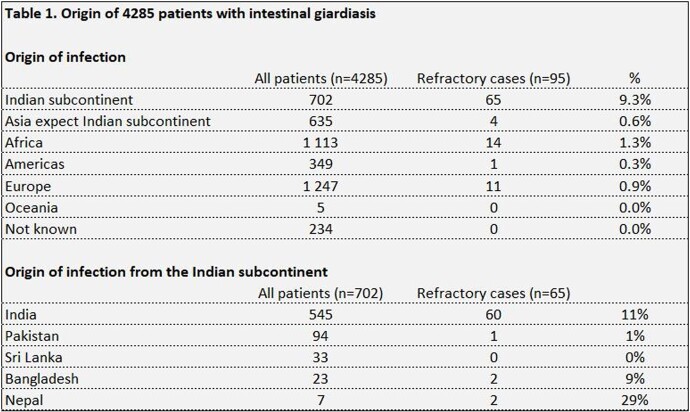

**Conclusion:**

The proportion of nitroimidazole-refractory giardiasis was ten times higher in those who had acquired the infection in India compared to other parts of the world, and should be taken into consideration.

**Disclosures:**

**All Authors**: No reported disclosures

